# 1171. Epidemiology of Old World Cutaneous Leishmaniasis among Members of the U.S. Military

**DOI:** 10.1093/ofid/ofac492.1008

**Published:** 2022-12-15

**Authors:** Brenan R Cebula, William D Porter, Glenn Wortmann, William R Byrne, Mark Polhemus, Kendall Billick, Wendy B Bernstein, Cliffton Hawkes, Mary Marovich, Naomi E Aronson

**Affiliations:** U.S. Military HIV Research Program, Walter Reed Army Institute of Research, Silver Spring, Maryland; U.S. Army Medical Center of Excellence, Fort Rucker, Alabama; MedStar Washington Hospital Center, Washington, District of Columbia; Walter Reed Army Medical Center, Frederick, Maryland; Coalition for Epidemic Preparedness Innovations, Syracuse, New York; University Health Network Toronto Western Hospital, Toronto, Ontario, Canada; Walter Reed National Military Medical Center, Bethesda, Maryland; Bon Secours Southside Medical Center, Petersburg, Virginia; National Institute of Allergy and Infectious Diseases, National Institutes of Health, Bethesda, Maryland; Uniformed Services University of the Health Sciences, Bethesda, Maryland

## Abstract

**Background:**

Cutaneous leishmaniasis (CL) is a threat to U.S. Military personnel as they deploy to endemic areas. As treatment may require evacuation, CL undermines operations. Elucidating the epidemiology of CL in this population is key for prevention.

**Methods:**

We retrospectively reviewed data from a CL sodium stibogluconate treatment trial at Walter Reed Army Medical Center, Washington DC. 412 military members with parasitologically confirmed CL and deployment to Southwest Asia from May 2002 - August 2004 enrolled. Subjects’ CL lesions were counted and measured. 334 subjects completed a risk survey. Given no control group, we used number of CL lesions (NL), total lesion area (TLA), and lesion location as outcomes to assess CL risks. Non-parametric tests and logistic regression were used as appropriate.

**Results:**

Permethrin treated bed net use was associated with lower NL (p = 0.000), TLA (p = 0.024), and odds of head/face lesion (OR 0.12, p = 0.047). Sleeping in a combat uniform was associated with lower TLA (p = 0.000) and odds of leg/foot lesion (OR 0.39, p = 0.023). Use of a permethrin treated uniform (p = 0.002) and N,N-diethyl-meta-toluamide (DEET) insect repellent at night (p = 0.046) were associated with lower NL. Noting unit members with similar lesions was associated with higher NL (p = 0.007) and odds of head/face lesion (OR 12.1, p = 0.019). National Guard status followed by Active Duty was associated with higher NL (p = 0.002) and TLA (p = 0.000) than Reserve. Sleeping in a building was associated with higher TLA (p = 0.008) and odds of arm lesion (OR 2.28, p = 0.016). Sleeping near animal burrows was associated with higher NL (p = 0.031). Sleeping on a cot (p = 0.006), certain ethnicities (p = 0.020) and certain military occupational specialties (p =0.038) were associated with higher TLA. Increasing age (p = 0.001) and years of service (p = 0.000) were positively correlated with TLA.

**Conclusion:**

This is the largest group of U.S. Military members with CL reported and provides insight into risks for CL which will guide preventive efforts to reduce the burden of illness in this population.

The opinions and assertions expressed herein are those of the author(s) and do not reflect the official policy or position of the Uniformed Services University of the Health Sciences, the Department of Defense, or the U.S. Government.

**Disclosures:**

**Naomi E. Aronson, M.D.**, Elsevier: Royalties as text editor, honoraria for chapter writing|UpToDate: royalties for writing chapters.

